# GSK3 Influences Social Preference and Anxiety-Related Behaviors during Social Interaction in a Mouse Model of Fragile X Syndrome and Autism

**DOI:** 10.1371/journal.pone.0009706

**Published:** 2010-03-16

**Authors:** Marjelo A. Mines, Christopher J. Yuskaitis, Margaret K. King, Eleonore Beurel, Richard S. Jope

**Affiliations:** Department of Psychiatry and Behavioral Neurobiology, University of Alabama at Birmingham, Birmingham, Alabama, United States of America; Case Western Reserve University, United States of America

## Abstract

**Background:**

Nearly 1% of children in the United States exhibit autism spectrum disorders, but causes and treatments remain to be identified. Mice with deletion of the *fragile X mental retardation 1* (*Fmr1*) gene are used to model autism because loss of *Fmr1* gene function causes Fragile X Syndrome (FXS) and many people with FXS exhibit autistic-like behaviors. Glycogen synthase kinase-3 (GSK3) is hyperactive in brains of *Fmr1* knockout mice, and inhibition of GSK3 by lithium administration ameliorates some behavioral impairment in these mice. We extended our studies of this association by testing whether GSK3 contributes to socialization behaviors. This used two mouse models with disrupted regulation of GSK3, *Fmr1* knockout mice and GSK3 knockin mice, in which inhibitory serines of the two isoforms of GSK3, GSK3α and GSK3β, are mutated to alanines, leaving GSK3 fully active.

**Methodology/Principal Findings:**

To assess sociability, test mice were introduced to a restrained stimulus mouse (S1) for 10 min, followed by introduction of a second restrained stimulus mouse (S2) for 10 min, which assesses social preference. *Fmr1* knockout and GSK3 knockin mice displayed no deficit in sociability with the S1 mouse, but unlike wild-type mice neither demonstrated social preference for the novel S2 mouse. *Fmr1* knockout mice displayed more anxiety-related behaviors during social interaction (grooming, rearing, and digging) than wild-type mice, which was ameliorated by inhibition of GSK3 with chronic lithium treatment.

**Conclusions/Significance:**

These results indicate that impaired inhibitory regulation of GSK3 in *Fmr1* knockout mice may contribute to some socialization deficits and that lithium treatment can ameliorate certain socialization impairments. As discussed in the present work, these results suggest a role for GSK3 in social behaviors and implicate inhibition of GSK3 as a potential therapeutic.

## Introduction

Autism Spectrum Disorders (ASDs) are a group of neurodevelopmental disorders characterized by deficits in social interactions and communication, and displays of repetitive behaviors. ASD is one of the most common behavioral disabilities diagnosed in children aged 3–5, 1 in 150 children in the United States was diagnosed with ASD in 2007 [1], [2], and the Centers for Disease Control and Prevention estimates that currently approximately 1 in 110 children in the United States has an ASD. Still-undefined combinations of genetic and environmental factors are thought to cause ASDs, and more effective treatments than those currently available are needed.

Animal models of ASDs are vital for studying the molecular mechanisms of the disorder and for developing effective therapeutics. Patients with Fragile X syndrome (FXS), caused by loss of function of the *fragile X mental retardation 1* (*Fmr1*) gene [3], often exhibit many of the symptoms commonly associated with ASDs, such as developmental delays, communication impairments, and anxiety [4]–[11]. These overlaps have led many investigators to conclude that *Fmr1* knockout mice provide a unique opportunity to identify interventions that affect autistic-like behaviors [12]–[14]. It is particularly relevant that *Fmr1* knockout mice have been found to display several deficits in social behaviors, including social dominance, social interest, social interaction, and social recognition, although differences in these behaviors have varied among the reports [12], [13], [15]–[19], as noted in the Discussion.

In *Fmr1* knockout mice, the FXS-related behaviors of sensitivity to audiogenic seizures, hyperactivity, and impaired passive avoidance memory were recently found to be effectively ameliorated by lithium [20], [21], an inhibitor of glycogen synthase kinase-3 (GSK3) that has been used in bipolar patients for many years [22]. Although GSK3 was first identified as an enzyme phosphorylating glycogen synthase, it has since been found to phosphorylate over 50 substrates [23]. Via substrate phosphorylation, GSK3 regulates many fundamental processes, including development, cell structure, microtubule dynamics, gene expression, and cell survival [24], [25]. GSK3 is a ubiquitous serine/threonine kinase that is present in mammals in two paralogs encoded by different genes that are commonly referred to as GSK3 isoforms, GSK3α and GSK3β [26]. Unlike many kinases that require a signal to be activated, GSK3 is constitutively partially active; therefore, signals impinging on GSK3 can either decrease or increase its activity. The most prevalent mechanism regulating the activity of GSK3 is inhibition by phosphorylation on serine-21 of GSK3α and serine-9 of GSK3β. Several kinases mediate this serine-phosphorylation, which greatly inhibits the activity of GSK3 [23]. A recently identified deficit in inhibitory serine-phosphorylation of GSK3 in *Fmr1* knockout mice raised the possibility that dysregulated GSK3 contributes to some of the behavioral phenotypes of these mice [20], [21]. The importance of inhibitory control of GSK3 can be studied using homozygous GSK3α^21A/21A^/β^9A/9A^ knockin mice, where the regulatory serines of both GSK3 isoforms are mutated to alanines [27]. These mutations maintain GSK3 maximally active, but importantly within the physiological range since both GSK3 isoforms are expressed at normal levels. Inhibitory serine-phosphorylation of GSK3 also is important for the action of lithium. Although lithium is a direct inhibitor of GSK3 [28], [29], at concentrations achieved in humans this is only a weak inhibition that is amplified by lithium-induced increases in inhibitory serine-phosphorylation of GSK3 [22], [30]. Thus, lithium treatment can reverse the deficit in serine-phosphorylated GSK3 in *Fmr1* knockout mice [20], [21], but this action is blocked in GSK3 knockin mice.

Since *Fmr1* knockout mice display social behavior deficits [13], [16], [17], [19], GSK3 is hyperactive in some brain regions of *Fmr1* knockout mice, and the GSK3 inhibitor lithium ameliorates several other impaired behaviors in *Fmr1* knockout mice [20], [21], the current study tested the hypothesis that impaired inhibitory regulation of GSK3 may contribute to deficits in sociability behaviors in *Fmr1* knockout mice. To do this, we tested whether administration of the GSK3 inhibitor lithium [28], [29], which increases inhibitory serine-phosphorylation of GSK3 ([20], [21], [30], restored normal social behaviors in *Fmr1* knockout mice, and tested whether GSK3 knockin mice, which completely lack the GSK3 inhibitory serine-phosphorylation, displayed any abnormal social behaviors that were similar to those of *Fmr1* knockout mice. The results indicate that GSK3 contributes to a subset of social behavioral deficits in *Fmr1* knockout mice, including impaired social preference and increased anxiety-related behaviors during social interaction.

## Results

### Sociability is similar in *Fmr1* knockout mice and wild-type mice and is increased by chronic lithium treatment

**Figure 1:**
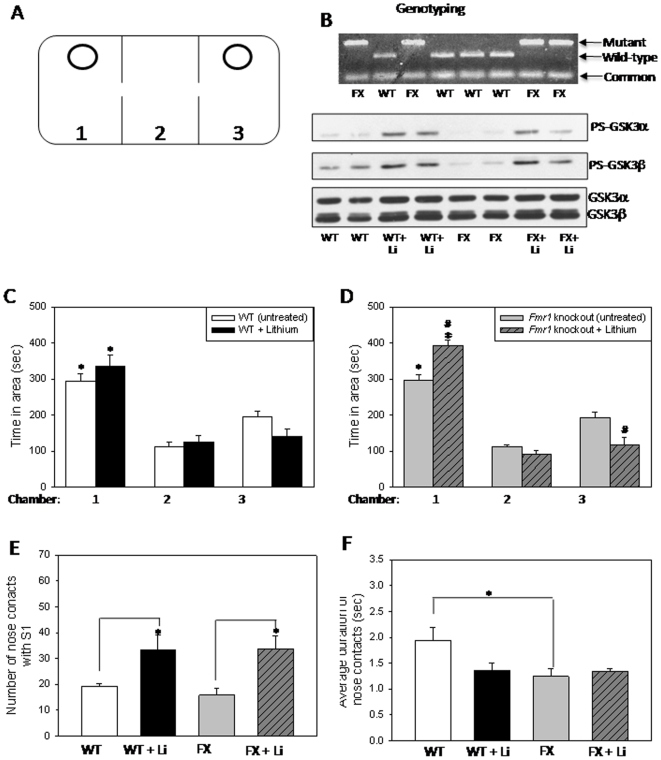
Influences of lithium treatment on the sociability of *Fmr1* knockout and wild-type mice. A. Diagram of the social interaction apparatus. The ovals represent the wire enclosures used to secure the stimulus mice, S1 in Chamber 1 and S2 in Chamber 3. The breaks in the center walls represent the circular openings allowing the mice to move between chambers. B. Representative image of genotyping results from wild-type and *Fmr1* knockout mice (top). Chronic lithium treatment increased inhibitory serine-phosphorylation of GSK3 (PS-GSK3) on serine-21 of GSK3α and on serine-9 of GSK3β in the hippocampus of wild-type and *Fmr1* knockout mice (bottom) as reported previously [20], [21]. C. Mean total time spent by wild-type mice in each chamber during the sociability period. Wild-type (WT) (n = 9) mice spent significantly more time in Chamber 1, the chamber with S1, compared to empty Chamber 2 or Chamber 3 with the wire enclosure only. Chronic lithium treatment of WT mice (n = 5) had no significant effect on sociability. * p<0.05 comparing time spent in Chamber 1 with time in Chambers 2 and 3. D. Mean total time spent by *Fmr1* knockout mice in each chamber during the sociability period. *Fmr1* knockout (FX) mice (n = 9) spent significantly more time in Chamber 1, the chamber with S1, compared to empty Chamber 2 or Chamber 3 with the wire enclosure only. FX mice (n = 6) chronically treated with lithium displayed a significant increase in time spent in Chamber 1, with S1, as compared to time in Chambers 2 or 3. Treated mice also displayed a significant increase in time spent in Chamber 1 and a significant decrease in time spent in Chamber 3, as compared to untreated controls. * p<0.05 comparing time spent in Chamber 1 with Chambers 2 and 3; # p<0.05 compared to untreated FX mice. E. The total number of nose contacts by the test mouse with S1 was equivalent for untreated WT and FX mice. Chronic lithium treatment significantly increased the number of nose contacts for both WT and FX mice. * p<0.05 compared to untreated group mates. F. The duration of nose contacts with S1 was lower in FX than WT mice. Lithium treatment had no effect on social interaction (defined as the average duration of nose contacts). * p<0.05 compared to untreated WT mice.

Wild-type and *Fmr1* knockout mice littermates (Fig. 1B) (∼3 months old) were individually placed in a social interaction apparatus to assess social behaviors, including sociability (preference for the chamber with an introduced mouse), social approach (number of nose contacts with an introduced mouse), and social interaction (duration of nose contacts). The sociability assessment involved the presence of a single wild-type stimulus mouse (S1) contained in a wire enclosure within Chamber 1 (Fig. 1). Chamber 2 was completely empty and Chamber 3 contained an empty wire cage enclosure. Both wild-type (Fig. 1C) and *Fmr1* knockout (Fig. 1D) mice displayed equivalent significant preferences for spending time in Chamber 1, with stimulus mouse (S1), compared with Chambers 2 or 3, and there was no significant interaction between genotype and chamber (F(2, 48)<0, P = 0.994). Chronic lithium treatment, which increased the inhibitory serine-phosphorylation of GSK3β in the brains of wild-type mice (Fig. 1B) [21], had no significant effect on sociability (Fig. 1C, black bars; F(1, 36)<0, P = 1) compared to untreated wild-type mice. In contrast, there was a significant interaction between lithium treatment and chamber among *Fmr1* knockout mice (Fig. 1D, F(2, 39) = 16.75, P<0.001). Lithium treatment promoted sociability in *Fmr1* knockout mice, significantly increasing the time *Fmr1* knockout mice spent in Chamber 1 with the stimulus mouse (Fig. 1D, slashed bars; F(2, 39) = 139.49, P<0.001), and significantly decreasing time spent in empty Chamber 3 (Fig. 1D, F(2, 39) = 139.49, P = 0.001) compared to untreated *Fmr1* knockout mice (Fig. 1D gray bars).

Sociability was further assessed by measuring social approach and social interaction. Social approach, quantitated as the number of nose contacts with the S1 stimulus mouse, was equivalent in untreated wild-type and *Fmr1* knockout mice (Fig. 1E, *t*(15) = 1.35, P = 0.197). Chronic lithium treatment significantly increased social approach, evident by increased number of nose contacts with S1 mouse, in wild-type mice (*t*(12) = 3.08, P = 0.01) and in *Fmr1* knockout mice (*t*(12) = 3.33, P = 0.006). This effect of lithium is unlikely due to increased activity of the mice because lithium administration did not alter the open field activity of wild-type mice and reduced activity of *Fmr1* knockout mice [20], [21]. The genotypes differed in social interaction, quantitated by the duration of nose contacts with the S1 mouse, which was significantly lower in *Fmr1* knockout mice than in wild-type mice (Fig. 1F, *t*(16) = 2.30, P = 0.035). Chronic lithium treatment had no significant effect on social interaction between wild-type mice and the S1 mouse (*t*(12) = 1.57, P = 0.142) or *Fmr1* knockout mice and the S1 mouse (*t*(12) = 0.462, P = 0.653). Thus, *Fmr1* knockout mice displayed a deficit in social interaction, and chronic lithium treatment increased sociability in *Fmr1* knockout mice and social approach in both groups of mice.

### *Fmr1* knockout mice exhibit impaired social interaction with a novel mouse, which is ameliorated by lithium treatment

**Figure 2:**
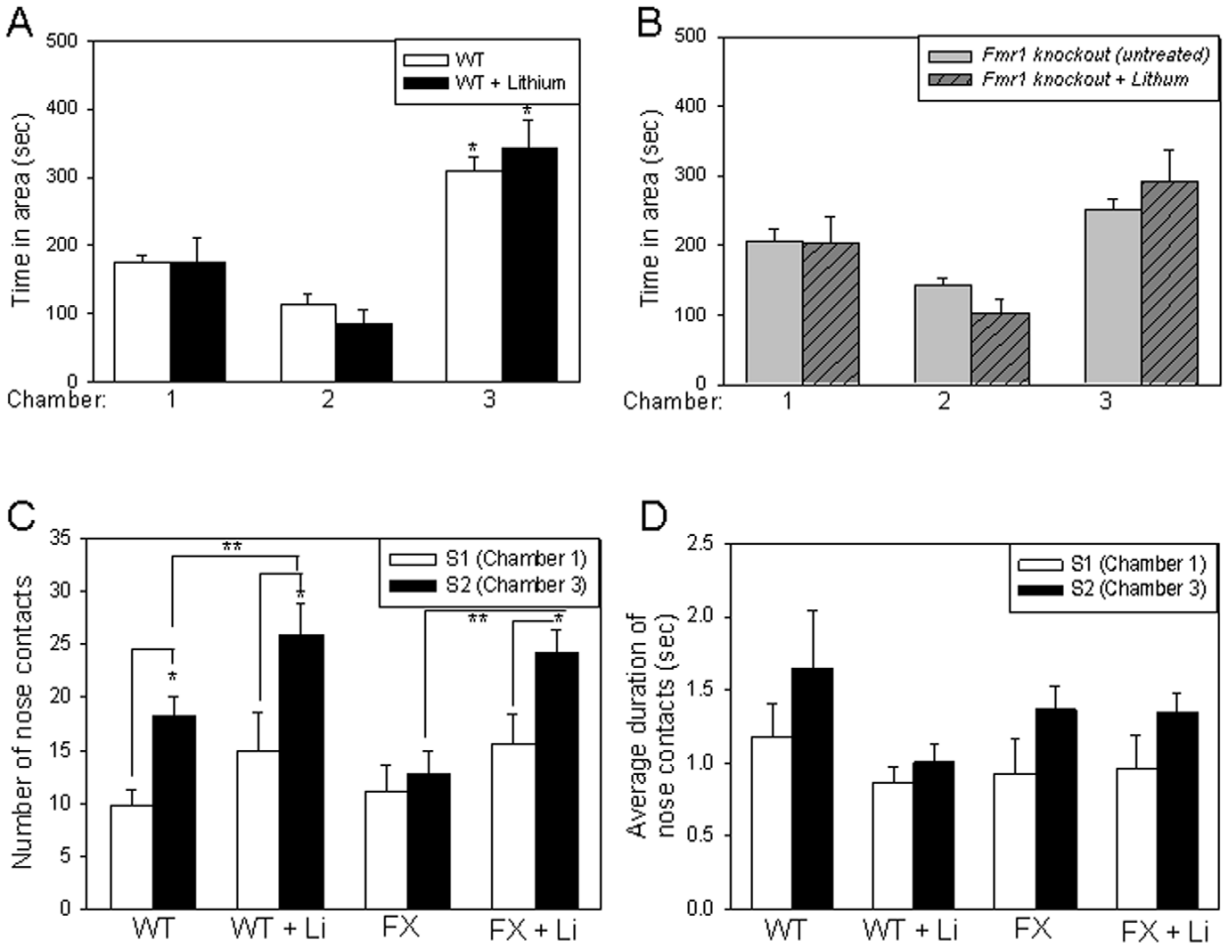
*Fmr1* knockout mice exhibit impairments in social preference. A. Mean total time spent by wild-type mice in each chamber during the social preference period. Wild-type (WT) mice (n = 9) spent significantly more time in Chamber 3 with the novel S2 mouse compared to empty Chamber 2, or to Chamber 1 with the familiar S1 mouse. Chronic lithium treatment did not significantly alter time spent in each chamber (n = 5). * p<0.05 comparing time spent in Chamber 3 with Chambers 1 and 2. B. Mean total time spent by *Fmr1* knockout mice in each chamber during the social preference period. There was not a significant difference in the amount of time *Fmr1* knockout (FX) mice (n = 9) spent in Chamber 3 with the novel mouse S2 and in Chamber 1 with the familiar mouse S1. Chronic lithium treatment did not significantly alter time spent in each chamber (n = 6). C. WT mice, but not FX mice, displayed a significant preference for nose contacts with S2, compared to S1. Chronic lithium treatment significantly increased the number of nose contacts with S2 by both WT and FX mice. * p<0.05 nose contacts with S2 compared with S1; ** p<0.05 compared to nose contacts with S2 by untreated group mates. D. The duration of nose contacts with S1 and S2 was not significantly different between WT and FX mice. Chronic lithium treatment had no significant effect on nose contact duration.

Social preference was measured by placing another stimulus mouse (S2) in Chamber 3 and comparing the interactions of wild-type and *Fmr1* knockout mice with the familiar S1 mouse and the novel S2 mouse. There was an overall significant interaction between genotype and chamber among wild-type and *Fmr1* knockout mice (F(2, 48) = 5.66, P = 0.006). As shown in Figure 2A (open bars), wild-type mice spent significantly more time in Chamber 3 with the novel S2 mouse than in Chamber 1 with the familiar S1 mouse or in the empty Chamber 2 (F(2, 36) = 50.40, P<0.001). Chronic lithium treatment caused no significant interaction between treatment and chamber and no significant change in sociability among wild-type mice (Fig. 2A, black bars; F(2, 36) = 0.95, P = 0.396). Untreated *Fmr1* knockout mice did not display a significant preference for the novel S2 mouse compared with the familiar S1 mouse, as there was no significant difference in the amount of time spent in Chamber 1 and Chamber 3 (Fig. 2B, gray bars; F(2, 39) = 19.77, P = 0.151). Thus, although *Fmr1* knockout mice displayed no deficits in sociability compared with wild-type mice during period 1, *Fmr1* knockout mice did not display a preference for the novel mouse during period 2, thereby suggesting a deficit in social preference. Chronic lithium treatment did not promote a significant interaction between treatment and chamber (Fig. 2B, F(2, 39) = 1.39, P = 0.261), and did not cause significant differences in the times that *Fmr1* knockout mice spent in Chamber 3 with the novel S2 mouse, compared to Chamber 1 with the familiar S1 mouse (Fig. 2B, slashed bars; F(2, 39) = 19.77, P = 0.232).

In social approach, as measured by the number of nose contacts with S1 or S2, there was no significant interaction between genotype and stimulus mouse (or chamber of interaction) among wild-type and *Fmr1* knockout mice (Fig. 2C, F(1, 32) = 2.98, P = 0.094). Wild-type mice, however, showed a significant preference for approaching S2 in Chamber 3, compared to S1 (F(1, 32) = 6.282, P = 0.005). Chronic lithium treatment of wild-type mice did not induce a significant interaction between treatment and stimulus mouse (or chamber of interaction; F(1, 24) = 0.257, P = 0.617), but caused a significant increase in the social approach of wild-type mice with S2, thereby increasing the number of nose contacts with S2 (F(1, 24) = 17.13, P = 0.030). Lithium treatment of *Fmr1* knockout mice did not induce any significant interaction between treatment and stimulus mouse (or chamber of interaction) (F(1, 26) = 2.024, P = 0.167). However, after lithium treatment *Fmr1* knockout mice displayed a significant increase in nose contacts with S2 compared to S1 (F(1, 26) = 4.24, P = 0.03). Thus, lithium appears to correct the deficit in social preference among *Fmr1* knockout mice, as a significant difference in social preference for S2 occurred after treatment.

There was no significant interaction between genotype and stimulus mouse (or chamber of interaction) in social interaction, the duration of nose contacts (Fig. 2D, F(1, 32) = 0, P = 0.952. Chronic lithium treatment of wild-type mice did not induce a significant interaction between treatment and stimulus mouse (or chamber of interaction), as shown in Fig. 2D (F(1, 24) = 0.274, P = 0.605). Lithium treatment of *Fmr1* knockout mice did not cause a significant difference in social interaction and did not induce a significant interaction between treatment and stimulus mouse (or chamber or interaction) (F(1, 26) = 0.01, P = 0.910).

### *Fmr1* knockout mice exhibit increased anxiety-related behaviors during social interaction

**Figure 3:**
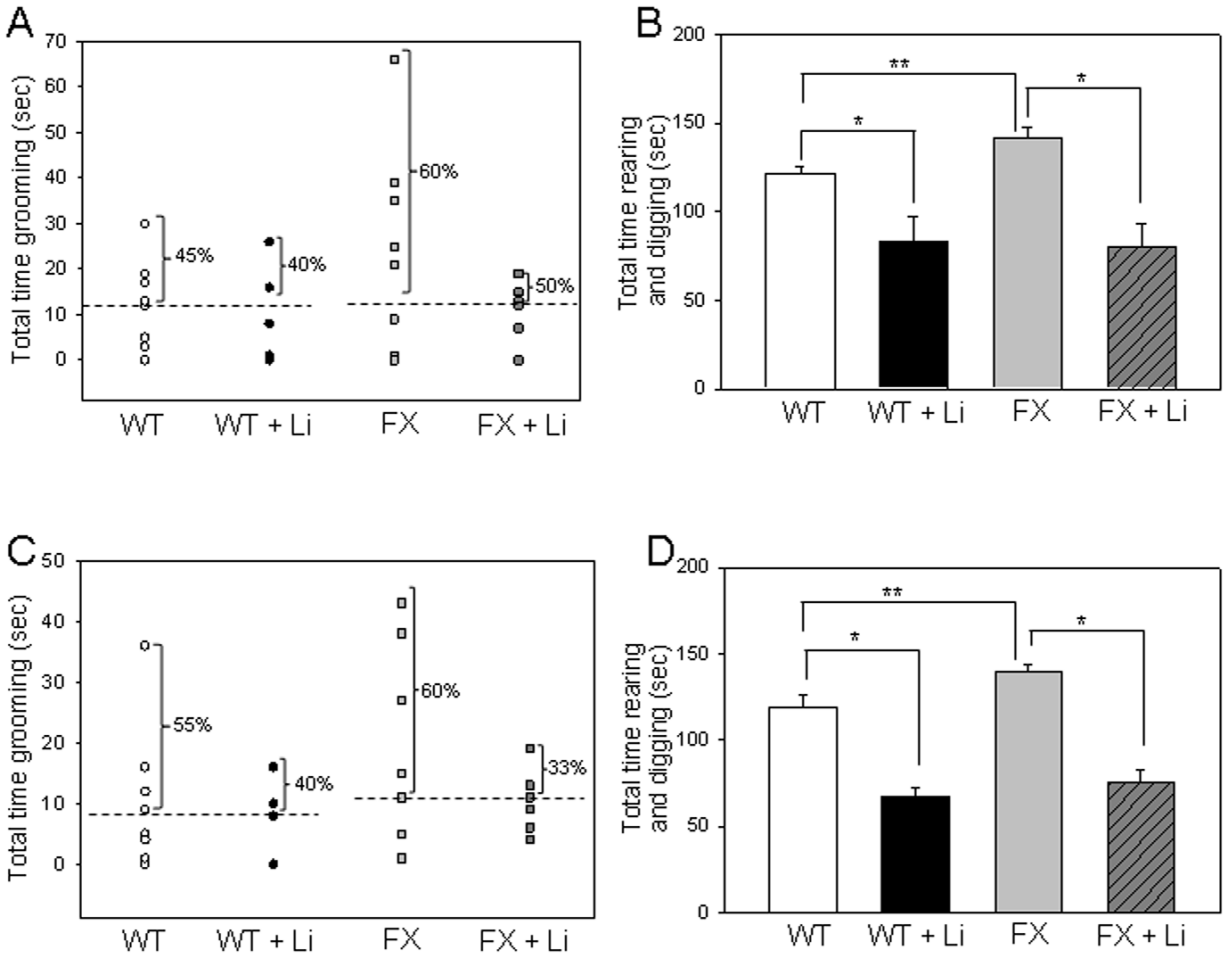
*Fmr1* knockout mice exhibit increased social anxiety-related behaviors during social interaction. A. During the sociability period, there was not a significant difference in grooming times of *Fmr1* knockout (FX) mice and wild-type (WT) mice. Chronic lithium treatment had no significant effect on WT grooming times but slightly decreased the percentage of FX mice with grooming times above the median time. Dashed lines represent median grooming times, 12 sec and 12.5 sec for WT and FX mice, respectively. B. FX mice spent significantly more time rearing and digging than WT mice during the sociability period. Chronic lithium treatment significantly decreased duration of rearing and digging behaviors in both groups of mice. *p<0.05 compared to untreated group mates; ** p<0.05 comparing untreated WT and FX mice. C. During the social preference period, there was not a significant difference in grooming times of *Fmr1* knockout (FX) mice and wild-type (WT) mice. Chronic lithium treatment had no significant effect on grooming times, but slightly decreased the percentage of WT and FX mice with grooming times above the median time. Dashed lines represent median grooming times, 8.5 sec and 11 sec for WT and FX mice, respectively. D. FX mice spent significantly more time rearing and digging than WT mice during the social preference period. Chronic lithium treatment significantly decreased duration of rearing and digging behaviors in both groups of mice. * p<0.05 compared to untreated group mates; ** p<0.05 comparing untreated WT and FX mice.

Anxiety-related behaviors during social interaction, including grooming, rearing and digging, were measured in each period of the tests. During the sociability period (Fig. 3A) and the social preference period (Fig. 3C), there were no significant differences in grooming behavior between untreated wild-type mice and *Fmr1* knockout mice. Comparing lithium treated wild-type mice to untreated wild-type mice, there was no significant effect of lithium treatment on grooming behavior (sociability: *t*(12) = 0.201, P = 0.844; preference: *t*(12) = 0.688, P = 0.505). *Fmr1* knockout mice treated with lithium also had no significant differences in grooming behaviors compared to untreated *Fmr1* knockout mice (sociability: *t*(13) = 1.14, P = 0.276; preference: *t*(13) = 1.580, P = 0.138). Despite the overall lack of significant difference between untreated wild-type mice and *Fmr1* knockout littermates, similarly to the results reported by McNaughton et al (2008), most evident was the much broader range of interindividual differences in the grooming times of *Fmr1* knockout mice compared with wild-type mice, and chronic lithium treatment reduced the interindividual variation in grooming times of *Fmr1* knockout mice.

During both the first sociability period (Fig. 3B) and the social preference period (Fig. 3D), *Fmr1* knockout mice spent significantly more time rearing and digging than wild-type mice (sociability: *t*(16) = 2.83, P = 0.012; preference: *t*(16) = 2.50, P = 0.024). During the first sociability period chronic lithium treatment reduced rearing and digging behaviors of wild-type mice compared to untreated wild-type mice (sociability: *t*(12) = 3.43, P = 0.005; preference: *t*(12) = 4.61, P = 0.000) and significantly reduced the rearing and digging times of *Fmr1* knockout mice compared to untreated *Fmr1* knockout mice (sociability: *t*(13) = 4.87, P = 0.000; preference.: *t*(13) = 9.19, P = 0.000). During the social preference period, lithium treatment caused a ∼55% decrease in time rearing and digging for the wild-type mice and a ∼54% decrease for the *Fmr1* knockout mice, eliminating the significant difference between the genotypes (Fig. 3D). These data demonstrate that *Fmr1* knockout mice exhibit more anxiety-related time rearing and digging behaviors during social interaction than wild-type mice that were reduced by chronic lithium treatment.

### GSK3 knockin mice display normal sociability behaviors

**Figure 4:**
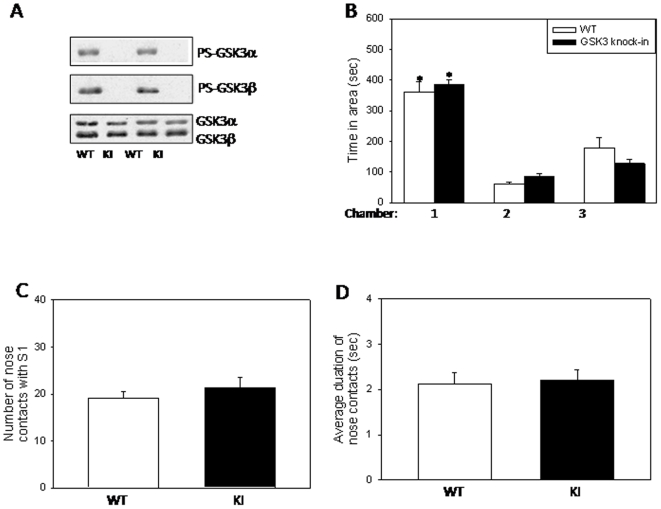
Sociability is not altered in GSK3 knockin mice. A. GSK3 knockin mice lack serine-phosphorylation of GSK3 (PS-GSK3) on serine-21 of GSK3α and on serine-9 of GSK3β, which is evident in wild-type mice, as reported previously [27], [38]. Total GSK3 levels are equivalent in GSK3 knockin mice and wild-type mice. B. Mean total time spent in each chamber during the sociability period. Wild-type (WT) (n = 7) mice and GSK3 knockin (KI) (n = 10) mice spent significantly more time in Chamber 1, the chamber with S1, compared to empty Chamber 2 or Chamber 3 with the wire enclosure only. * p<0.05 comparing time spent in Chamber 1 with Chambers 2 and 3. C. Social approach, the total number of nose contacts by the test mouse with S1, was equivalent for untreated WT and KI mice. D. There was no difference in social interaction between WT and KI mice.

To test if the decreased inhibitory serine-phosphorylation of GSK3 in *Fmr1* knockout mice is sufficient to cause the observed deficits in socialization, social behaviors were measured with GSK3 knockin mice, which have S21A-GSK3α and S9A-GSK3β mutations, thereby disabling the inhibitory serine phosphorylation of GSK3 (Fig. 4A). During the sociability period, there was no significant interaction between genotype and chamber (F(2, 45) = 2.521, P = 0.092). GSK3 knockin mice spent similar times as wild-type mice in each chamber, with both genotypes spending more time in Chamber 1 containing the S1 mouse than in Chamber 3 containing only the wire enclosure (Fig. 4B, WT: F(2, 45) = 117.29, P<0.001, GSK3 knockin: F(2, 45) = 117.29, P<0.001). There were also no significant differences between wild-type mice and GSK3 knockin mice in social approach (the number of nose contacts, Fig. 4C, *t*(15) = 0.746, P = 0.467) or social interaction (average duration of nose contacts, Fig. 4D, *t*(15) = 0.220, P = 0.829).

### GSK3 knockin mice exhibit impaired social preference

**Figure 5:**
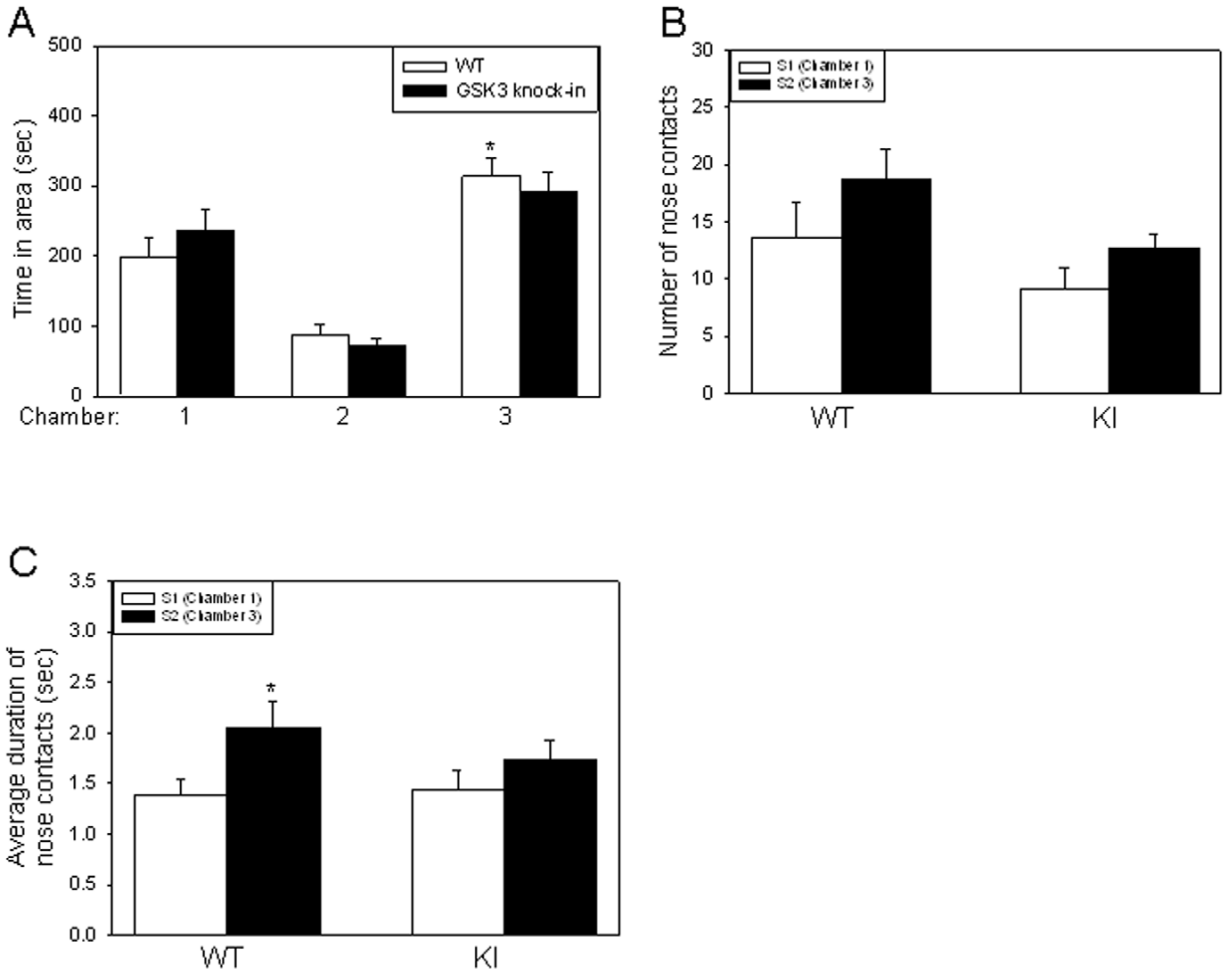
GSK3 knockin mice exhibit impairments in social preference. A. Mean total time spent in each chamber during the social preference period. Wild-type (WT) mice (n = 11), but not GSK3 knockin (KI) mice (n = 10), spent significantly more time in Chamber 3 with the novel S2 mouse compared to empty Chamber 2, or to Chamber 1 with the familiar (S1) mouse. * p<0.05 comparing time spent in Chamber 3 with Chambers 1 and 2. B. GSK3 KI mice display deficits in social approach preference, as indicated by no significant difference in the number of nose contacts with S1 or S2. C. The duration of nose contacts with S2 was significantly greater than with S1 for WT, but not GSK3 KI, mice. * p<0.05 comparing WT duration of nose contacts with S1 and S2.

Since *Fmr1* knockout mice exhibited impaired social preference (Fig. 2), we tested if this was also altered in GSK3 knockin mice. There was no significant interaction between genotype and chamber during this testing period (F(2, 57) = 0.985, P = 0.380). Wild-type mice spent significantly more time in Chamber 3 with the novel S2 mouse than in Chamber 1 with the familiar mouse (Fig. 5A, F(2, 57) = 45.620, P<0.001). In contrast, there was no significant difference in the amount of time that GSK3 knockin mice spent in Chamber 3 with the novel S2 mouse and in Chamber 1 with the familiar S1 mouse (Fig. 5A, F(2, 57) = 45.620, P = 0.111).

Assessment of social approach revealed that there was no significant interaction between genotype and stimulus mouse (or chamber of interaction, Fig. 5B, F(1, 34) = 0.111, P = 0.741). GSK3 knockin mice did not exhibit a significant preference for approaching S2 in Chamber 3 compared with S1 in Chamber 1 (F(1, 34) = 3.566, P = 0.266). There was also no significant interaction between genotype and stimulus mouse (or chamber or interaction) when assessing social interaction (the duration of nose contacts) behavior (Fig. 5C, F(1, 34) = 0.788, P = 0.381). Wild-type mice displayed a preference for social interaction with S2 in Chamber 3, as demonstrated by the increased duration of nose contacts with S2 compared with S1 (Fig. 5C, F(1, 34,) = 5.336, P = 0.035). GSK3 knockin mice displayed no preference for the S2 stimulus mouse in Chamber 3 as indicated by no significant difference between social interaction with S1 and S2 (F(1, 34) = 5.336, P = 0.309). These results are similar to those observed with the *Fmr1* knockout mice, suggesting a possible correlation between less inhibitory serine phosphorylation of GSK3 and impaired social preference for a novel stimulus mouse.

### GSK3 knockin mice display little anxiety-related behaviors during social interaction

**Figure 6:**
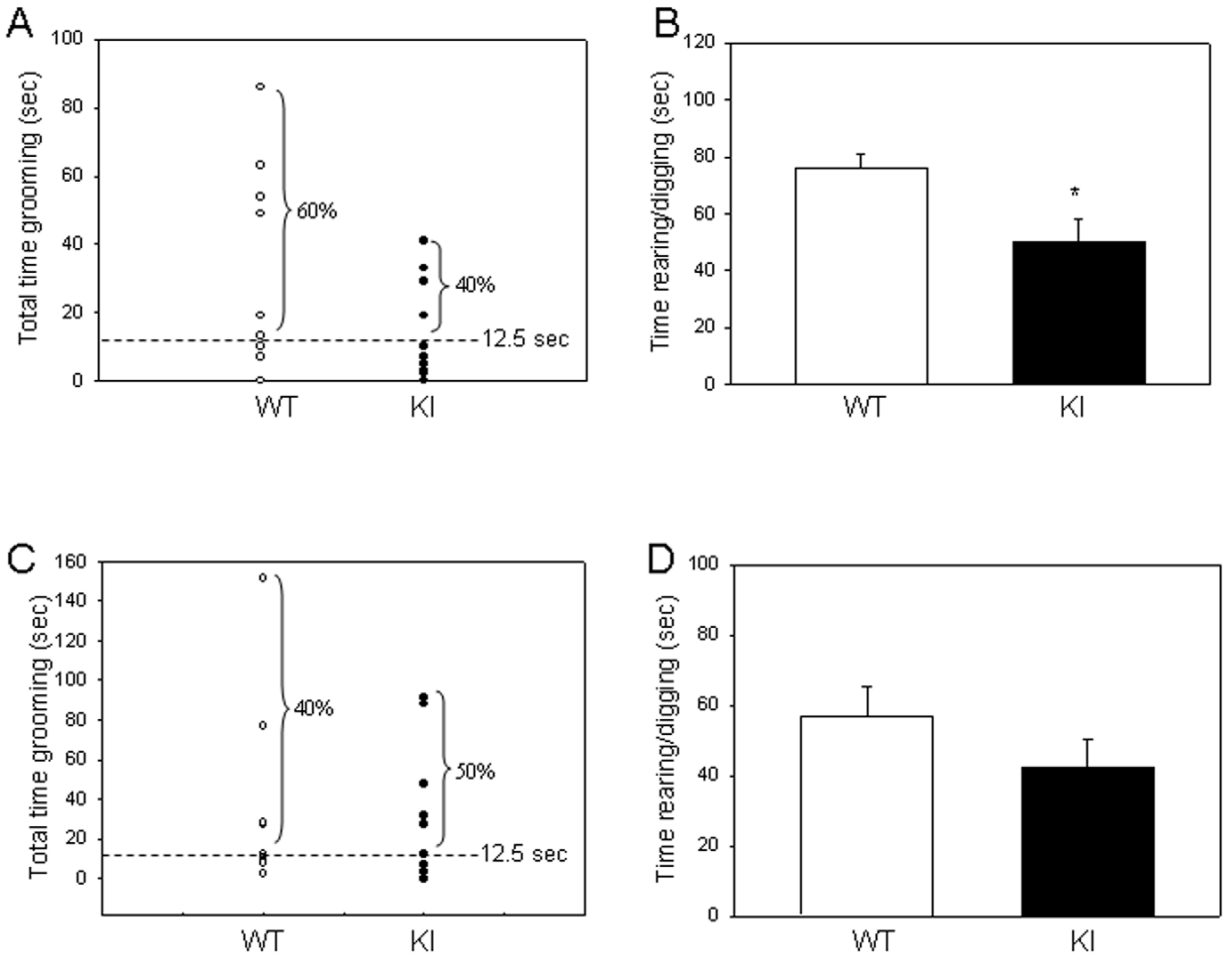
Anxiety-related behaviors during social interaction in GSK3 knockin mice. A. During the sociability period, there was not a significant difference in grooming times of GSK3 knockin (KI) mice and wild-type (WT) mice, but the percentage of wild-type (WT) mice with grooming times above the median was higher than GSK3 knockin (KI) mice. Dashed lines represent median grooming times, 12.5 sec. B. GSK3 KI mice spent significantly less time rearing and digging than WT mice during the sociability period. * p<0.05 compared to untreated WT mice. C. During the social preference period, there was not a significant difference in grooming times of GSK3 knockin (KI) mice and wild-type (WT) mice, but the percentage of KI mice with grooming times above the median was slightly higher than WT controls. Dashed lines represent median grooming times, 12.5 sec. D. WT and GSK3 KI mice spent similar times rearing and digging during the social preference period.

*Fmr1* knockout mice exhibited greater anxiety-related behaviors during social interaction than wild-type mice. Using the same testing paradigm, we compared GSK3 knockin mice and wild-type mice. There was no significant difference in time spent grooming between wild-type and GSK3 knockin mice during the sociability period with only S1 (Fig. 6A, *t*(17) = 1.73, P = 0.101), or during the social preference period with S1 and S2 (Fig. 6C, *t*(17) = 0.27, P = 0.790). During the first sociability period, wild-type mice spent 66% more time rearing and digging than the GSK3 knockin mice (Fig. 6B, *t*(15) = 2.46, P = 0.027). During the social preference period, there was no significant difference in rearing and digging times between wild-type and GSK3 knockin mice (Fig. 6D, *t*(17) = 1.28, P = 0.218). Collectively, there is little difference in anxiety-related behaviors during social interaction in the GSK3 knockin mice compared to wild-type mice.

## Discussion

Impairments in sociability have been identified previously in *Fmr1* knockout mice that appear to model some of the deficits in social interactions characteristic of FXS and ASDs [12], [13], [15]–[19]. Since there is a need for more effective interventions for impaired social behaviors than those currently available, we tested the efficacy of lithium, an inhibitor of GSK3, because GSK3 is hyperactive in *Fmr1* knockout mouse brain and lithium ameliorates several other abnormal behaviors in *Fmr1* knockout mice, including hyperactivity, sensitivity to audiogenic seizures, and impaired performance in a passive avoidance learning task [20], [21]. Conversely, we tested whether GSK3 knockin mice expressing hyperactive GSK3 [27] displayed any sociability impairments resembling those of *Fmr1* knockout mice. The results obtained from these two experimental approaches indicate that GSK3 may contribute to some aspects of social interaction impairments, supporting the possibility that lithium, in combination with other interventions, may contribute to improved sociability behaviors in patients with FXS or ASDs.

Although identifying differences in social behavior between *Fmr1* knockout and wild-type mice was not the main goal of this study, as this has been addressed by several previous reports [12], [13], [15]–[19], this aspect provided the necessary background for our principal goal of testing whether social behaviors are influenced by changes in the serine-phosphorylation of GSK3, which regulates its activity. Upon examination of the results from this and previous sociability studies of *Fmr1* knockout mice, two aspects stand out, (i) the limited magnitude, and (ii) the variability, in reported social behavior differences between *Fmr1* knockout and wild-type mice. First, the most conspicuous aspect of social behavior in *Fmr1* knockout mice may be that differences from wild-type mice are fairly subtle. This is in marked contrast to the severity of deficits in social behaviors that are often evident in FXS and ASDs [4]–[11], [13]. This subtle impairment of sociability behaviors matches the similarly modest deficits in learning tasks displayed by *Fmr1* knockout mice, although this is also a major characteristic of FXS [21], [31], [32]. In contrast, *Fmr1* knockout mice appear to be an excellent model for the hyperactivity and seizure susceptibility aspects of FXS. Second, it is also evident that there is not a single aspect of altered sociability behavior in *Fmr1* knockout mice that has been found to be a robustly reproducible phenotype, but instead somewhat different characteristics have been identified in each report, likely in part due to the subtlety of their social behavioral deficits. Spencer *et al.* (2005) reported that *Fmr1* knockout mice exhibited increased social approach behavior, decreased anxiety-like behaviors, and no difference in sociability, compared to wild-type mice [16]. Extending their studies, Spencer *et al.* (2008) reported that *Fmr1* knockout mice had increased locomotor activity, displayed less anxiety-like responses to novel food, and exhibited increased social approach (or an increase in the number of social approaches toward a novel cage-mate) than their wild-type counterparts [18]. Expression of human FMRP, using a yeast-artificial chromosome transgenic mouse model, corrected several altered behaviors, including normalized locomotor activity, normalized anxiety-like responses to novel food, and normalized social approach [18]. Overall, the findings of this group supported the conclusion that *Fmr1* expression regulates certain social behaviors. Mineur *et al.* (2002) reported increased locomotor activity, decreased rearing, and decreased grooming behaviors among *Fmr1* knockout mice in the open field test, compared to wild-type mice [15]. Using the partition interaction test, with ovariectomized C3H female mice as the stimulus, this group reported that *Fmr1* knockout mice exhibited deficient social interaction and impaired social preference [17]. McNaughton *et al.* (2008) used the three-chambered social interaction box and matched background and aged male mice as the stimulus and reported no deficit in sociability, social approach, or social preference in *Fmr1* knockout mice [19]. Liu & Smith (2009) used the three-chambered social behavior box and matched background, weight, and aged male mice as the stimulus and reported decreased social approach, affected social preference, and increased social anxiety in *Fmr1* knockout mice [13]. Overall, a clear consensus has not been reached on most sociability behaviors displayed by *Fmr1* knockout mice. Likely causes for these variable results among laboratories have been discussed in detail previously, focused primarily on methodological variables [12], [13], [19], [33]. For example, as detailed in those reports, the sociability studies have varied in the background of the mice studied, including C57Bl/6J X FVB/NJ [19], FVB/NJ [12], [13], and C57Bl/6J [12], [15]–[18], the time of day tests were administered (during the light cycle: [13], [16], [18]; or during the dark cycle: [19], the age of the mice examined (2–3 months old: [12], [13]; 3–4 months old: [12], [15]–[18]; this study; 10–12 months old: [19]), and other variable methods. These and other experimental variations discussed previously [13], [19] likely contribute to variable findings among laboratories along with the subtlety of the social behavioral impairments in the *Fmr1* knockout mice. Thus, although the *Fmr1* knockout mice have limitations in applications to studies of possible therapeutic interventions for sociability aspects of FXS and ADS, they remain the best model available until others are developed.

The present study, using C57Bl/6J *Fmr1* knockout mice and the three-chambered social behavior apparatus during the dark cycle, focused on testing whether GSK3 influences social behavior in *Fmr1* knockout mice. GSK3 was studied because inhibitory serine-phosphorylation of GSK3 is impaired in *Fmr1* knockout mouse brains, and administration of GSK3 inhibitors controls other behavior abnormalities in *Fmr1* knockout mice [20], [21]. The effects of GSK3 on social behaviors were studied two ways. First, lithium, a selective GSK3 inhibitor, was administered to wild-type and *Fmr1* knockout mice to test if it altered social behaviors. Lithium also has other actions [34] that could conceivably contribute to its effects on social behaviors. However, GSK3 is the most likely target because lithium inhibits GSK3 in the brains of wild-type and *Fmr1* knockout mice [20], [21], other GSK3 inhibitors reduce audiogenic seizures and hyperactivity in *Fmr1* knockout mice [20], and GSK3 is the target of lithium's therapeutic actions in other disorders [34], [35]. Second, we tested whether impaired inhibitory control of GSK3 was sufficient to alter social behaviors similarly to *Fmr1* knockout mice by using GSK3 knockin mice, in which GSK3 cannot be inhibited by serine-phosphorylation.

As in previous reports, only certain social behaviors were altered in *Fmr1* knockout mice. During the sociability period, wild-type and *Fmr1* knockout mice spent similarly greater times in the chamber with the S1 stimulus mouse than in the empty chambers. This confirms previous reports that *Fmr1* knockout mice, like wild-type mice, spend more time with another mouse than time alone ([12], [13], [19]. Introduction of a novel S2 stimulus mouse revealed a deficit in social preference in *Fmr1* knockout mice, as they spent similar times with the novel S2 and with the familiar S1 mouse, whereas wild-type mice preferred the novel mouse. Some, but not all, previous studies have reported impaired social preference of *Fmr1* knockout mice [12], [16], [19], with variations likely due to methodological and mouse strain differences [33]. Just as the effects of lack of FMRP on social behavior are modest, so too was the influence of altered GSK3 activity. Interestingly, GSK3 knockin mice displayed similar behaviors as *Fmr1* knockout mice, displaying equivalent sociability as wild-type mice with a single stimulus mouse, but showing no preference for a novel mouse over a familiar mouse. However, interpretation of the results with the GSK3 knockin mice is limited by the impossibility of studying littermates because of the double mutation in the homozygous GSK3 knockin mice, which through environmental factors in the home-cage could affect sociability. Nonetheless, it is interesting that *Fmr1* knockout mice and GSK3 knockin mice share the phenotype of impaired social preference. This impaired preference for a novel mouse could indicate an inability to discriminate between familiar and novel mice, reduced interest in novelty, or other causes, but the interesting point is that this behavioral difference was shared between the *Fmr1* knockout mice and the GSK3 knockin mice, which both share increased GSK3 activation levels in the brain. Taken together, these findings indicate that GSK3 does not markedly influence sociability but impaired inhibitory serine-phosphorylation of GSK3 in both *Fmr1* knockout mice and in GSK3 knockin mice is associated with a lack of social preference for a novel mouse.

As with sociability and social preference, there were also differential alterations in social approach in *Fmr1* knockout mice and in GSK3 knockin mice. Social approach in the presence of a single stimulus mouse was not altered in *Fmr1* knockout mice, but they displayed a significant deficit in social approach to a novel mouse during the social preference phase, thereby not preferring the novel S2. These results are similar to previous reports that *Fmr1* knockout mice displayed less social approach behavior with a novel mouse than wild-type mice [13], [17]. Like *Fmr1* knockout mice, social approach also was unaltered in GSK3 knockin mice in the presence of a single stimulus mouse, and GSK3 knockin mice approached a novel second mouse less than did wild-type mice. Thus, less inhibitory serine-phosphorylation of GSK3 in both *Fmr1* knockout mice and GSK3 knockin mice is associated with lack of preferential social approach with a novel mouse. Furthermore, lithium treatment increased social approach in both *Fmr1* knockout and wild-type mice. This is not due to increased locomotor activity since lithium reduces open field activity in *Fmr1* knockout mice and does not alter open field activity of wild-type mice [20], [21]. These results suggest that lower inhibitory serine-phosphorylation of GSK3 contributes to impaired displays of social preference.

Social avoidance among FXS and ASD patients may be partly due to social anxiety experienced in social settings [33]. *Fmr1* knockout mice displayed heightened anxiety-related behaviors during social interaction, as a higher percentage of *Fmr1* knockout mice than wild-type littermates displayed greater grooming times than the group median in both the sociability period and the social preference period, and spent more time rearing and digging. This is similar to the report of McNaughton *et al*. (2008) that a higher percentage of FX mice exhibited grooming times higher than the median in the presence of a stimulus mouse [19], and a report of *Fmr1* knockout mice exhibiting lower center mirror ratio and decreased win percentages in the dominance tube test [16], which indicated increased anxiety in *Fmr1* knockout mice. However, others found no elevation in social anxiety in *Fmr1* knockout mice [15], [16], [36]. The increased anxiety-related behaviors during social interaction in *Fmr1* knockout mice was not reflected in GSK3 knockin mice, indicating that impaired serine-phosphorylation of GSK3 is not sufficient to increase anxiety-related behaviors during social interaction. Nevertheless, inhibition of GSK3 by lithium administration reduced anxiety-related behaviors during social interaction in both wild-type and *Fmr1* knockout mice during both the sociability and social preference periods of testing. This suggests that although dysregulated GSK3 alone is not sufficient to cause anxiety-related behavior during social interaction, it can be reduced by inhibition of GSK3 with lithium in *Fmr1* knockout mice.

In summary, this study found that both *Fmr1* knockout mice and GSK3 knockin mice display normal sociability with a single stimulus mouse but do not display preference for a novel mouse. Chronic lithium treatment modestly increased sociability and more effectively reduced anxiety-related behaviors during social interaction in *Fmr1* knockout mice. Notably, a recent feasibility trial of lithium in patients with FXS noted improvements in several behaviors [37]. These findings provide the first identification of links between GSK3 and social behaviors and suggest that dysregulated GSK3 may contribute to some of the social impairments associated with loss of FMRP and that these might be partially remedied by lithium administration, also supporting the utility of *Fmr1* knockout as a means to identify mechanisms underlying social impairments common among ASD and FXS patients and for exploration of therapeutic interventions that may enhance social interactions.

## Materials and Methods

### Animals

This study used adult, male C57Bl/6J littermates, ∼3 months of age, with or without a disruption of the *Fmr1* gene (originally kindly provided by Dr. W. Greenough, University of Illinois), or homozygous GSK3α^21A/21A^/β^9A/9A^ knockin mice (hereafter referred to as GSK3 knockin mice; originally kindly provided with matched controls by Dr. D. Alessi, University of Dundee) and matched wild-type mice. The *Fmr1* knockout mice were generated by breeding male C57BL/6J hemizygous *Fmr1* knockout mice and female C57BL/6J heterozygous *Fmr1* knockout mice to generate male homozygous *Fmr1* knockout mice and wild-type littermates. Genotype was confirmed by PCR using the Jackson Laboratory protocol for genotyping *Fmr1* mice. The following primers were used: mutant 5′-CACGAGACTAGTGAGACGTG-3′, wild-type 5′-TGTGATAGAATATGCAGCATGTGA-3′, common 5′-CTTCTGGCACCTCCAGCTT -3′. Wild-type mice produce amplicon products at 131 base pairs, *Fmr1* knockout mice produce amplicon products at 400 base pairs, and heterozygous mice produce amplicons at 131 and 400 base pairs. GSK3 knockin mice and matched wild-type mice of mixed C57Bl6, Balb/c, and Ba11 background [27] were generated by continuous inter-breeding of homozygous knockin mice and wild-type mice of the same background. The GSK3 knockin mice contain serine-to-alanine mutations in the regulatory serines of both GSK3 isoforms, S21A-GSK3α and S9A-GSK3β [27]. These mutations disable the inhibitory serine phosphorylation of GSK3, but both isoforms are expressed at normal levels so GSK3 retains maximal activities within the normal physiological range. GSK3 knockin mice reproduce and develop normally and no overt phenotype has been reported. For chronic lithium treatment, mice were given *ad libitum* water and saline (to prevent hyponatremia caused by lithium-induced increased excretion of sodium) and were fed pelleted chow containing 0.2% lithium carbonate (Teklad, Madison, WI) for three weeks, as previously described [30], [38]. All mice were housed and treated in accordance with National Institutes of Health guidelines and procedures with mice were approved by the University of Alabama at Birmingham Institutional Animal Care and Use Committee.

### Behavior apparatus

A social interaction apparatus was used to assess sociability and social preference of mice, as previously described [19]. The apparatus (Fig. 1A) is a rectangular, transparent, Plexiglas box divided by Plexiglas walls into three equal sized compartments (24 cm length, 19 cm width, 19 cm height). Circular holes in the Plexiglas walls provide access between the chambers. The floor of the box was covered with a thin layer of bedding. All tests were performed during the dark cycle with a 25 W red light bulb. Sessions were recorded using a JVC mini camcorder on a MX 600 Tripod. The wire cages to house the stimulus mice were inverted, circular pencil holders with bars spaced 1 cm apart (11 cm height, 10 cm diameter; Galaxy Cup, Spectrum Diversified Designs, Inc., Streetsboro, OH) and were present in each chamber at all times. A clear, glass beaker was placed on top of each wire cage to prevent climbing. Between tests the apparatus was thoroughly washed with 70% ethanol and the bedding was changed.

### Behavior testing

*Habituation*: Test mice were individually allowed to freely explore the entire apparatus for 25 min the night prior to testing. Testing was completed in a single 25 min session comprised of three periods. Stimulus mice are confined to the small wire cages for 30 min each day for 5 days before the start of the test.

*Rehabituation* (5 min): The test mouse was placed in the center chamber and allowed to freely explore the apparatus. The mouse was never handled again until the conclusion of the test. When stimulus mice were to be placed in a chamber, the test mouse was gently coerced into the center chamber and confined from entering the side chambers with 3×5 index cards covering the inter-chamber openings.

*Sociability* (10 min): The test mouse was moved to the center Chamber 2 with the connecting holes blocked. An unfamiliar ∼3 month old male wild-type stimulus mouse 1 (S1) was placed in the wire enclosure in Chamber 1, the connecting holes to both Chambers 1 and 3 were opened, and the test mouse was allowed to explore the entire apparatus for 10 min. This period assesses socialization of the test mouse with the confined S1 mouse.

*Social Preference* (10 min): The test mouse was moved to the center Chamber 2 with the connecting holes blocked. A second, unfamiliar, wild-type stimulus mouse (S2) was placed in the wire enclosure in Chamber 3 while S1 remained in the wire enclosure in Chamber 1, the connecting holes were opened, and the test mouse was allowed to explore the entire apparatus for 10 min. This period assesses if the test mouse prefers to socialize more with the novel S2 mouse than the familiar S1 mouse.

### Data analysis

Each session was videotaped and videos were analyzed by pre-trained investigators blind to the test mouse genotype and treatment. Videos were quantitated for time spent in each chamber, nose contact number (any engagement between the nose of the test mouse and the confined stimulus mouse) and nose contact duration (time from initiation to disengagement of nose contacts), and cumulative time spent grooming, rearing, and digging. Face washing and body grooming times were combined for total grooming behavior times. Data were analyzed by two-way ANOVA (Holm-Sidak *posthoc* test) with genotype and chamber as factors or treatment and chamber as factors, or by Student's t-test (for social approach [number of nose contacts with an introduced mouse] and social interaction [duration of nose contacts with an introduced mouse] measurements in the sociability period and anxiety-like behaviors) to determine statistically significant differences among groups.
